# Association of Human Eastern Equine Encephalitis With Precipitation Levels in Massachusetts

**DOI:** 10.1001/jamanetworkopen.2019.20261

**Published:** 2020-01-31

**Authors:** Leonard A. Mermel

**Affiliations:** 1 Department of Medicine, Warren Alpert Medical School, Brown University, Providence, Rhode Island; 2 Division of Infectious Diseases, Rhode Island Hospital, Providence; 3 Department of Epidemiology and Infection Control, Rhode Island Hospital, Providence

## Abstract

This cross-sectional study examines the association of annual precipitation levels with annual rates of human Eastern equine encephalitis infection in Massachusetts.

## Introduction

From 2009 through 2018, only 3 to 15 cases of Eastern equine encephalitis (EEE) virus neuroinvasive disease in the United States were reported to the Centers for Disease Control and Prevention annually.^1^ The enzootic cycle of the EEE virus involves *Culiseta melanura* mosquitos and passerine birds inhabiting freshwater swamps. The likelihood of human EEE infection is associated with ecosystems where *C melanura* thrive. The spread of the EEE virus from infected birds to humans requires the presence of other mosquitos that feed on both birds and humans (ie, bridge vectors), such as *Coquillettidia pertubans*, *Aedes sollicitans*, or *Ochlerotatus canadensis*. Increased precipitation throughout the year maintains *C melanura* larval habitat.^2^ Additionally, global warming may amplify the EEE virus life cycle^3^ and expand EEE virus geographic distribution.^4^ I hypothesized that higher than usual precipitation during the preceding year from a midsummer month would be associated with increased human EEE infections.

## Methods

This cross-sectional study used the Standard Precipitation Index (SPI) in the 6 regions of Massachusetts. The SPI measures the probability of precipitation over a given period of time based on long-term precipitation records. The lower the SPI, the dryer the conditions, and the higher the SPI, the wetter the conditions compared with long-term historical data. These data were obtained from the Massachusetts Department of Conservation and Recreation. The number of human EEE infections each year from January 1, 1999, until December 1, 2019, was obtained from the Massachusetts Department of Public Health. The exposure variable was the SPI in the 6 regions of Massachusetts during 12 months, calculated from July of each year (eg, the SPI of 2019 includes August 1, 2018, through July 31, 2019), and the outcome variable was the number of human EEE cases in Massachusetts. Two-sided Fisher exact test was used to determine significance at *P* < .05. An a priori study size calculation was not performed. Statistical analysis was performed using VassarStats (Richard Lowry). Data were analyzed from September 18 to 20, 2019.

Per the Common Rule, this study did not require ethics board review because no protected health data of any kind were accessed or reviewed. The Strengthening the Reporting of Observational Studies in Epidemiology (STROBE) reporting guideline for cross-sectional studies was followed.

## Results

There were 38 human EEE cases reported to the Massachusetts Department of Public Health from 1999 to 2019 (Table). At least 1 human EEE case occurred in Massachusetts in 10 of 14 years when the SPI was higher than 1.0 in 1 or more regions of Massachusetts. However, a human EEE case occurred in only 1 of 7 years when the SPI was lower than 1.0 in all 6 regions of Massachusetts (relative risk, 3.0; 95% CI, 1.2-7.2; *P* = .02). Of note, 2019 was the first year in the last 20 years that the 12-month SPI measured in July was higher than 2.0 for all Massachusetts regions except 1 region (the Cape and Islands), and there were 12 human EEE cases in 2019.

**Table.** Human EEE Cases and 12-Month SPI From July of Each Year in Massachusetts

Calendar Year	Regional SPI^a^						Human EEE Cases, No.
	Western	Connecticut River	Central	Northeast	Southeast	Cape and Islands	
1999^b^	−0.75	−1.31	−1.26	−0.87	−0.32	−1.69	0
2000	1.56	2.07	0.89	1.20	1.24	−0.19	1
2001^b^	−0.32	−0.20	−0.41	0.37	0.67	−1.18	1
2002^b^	−1.27	−1.41	−1.55	−0.98	−0.96	−1.44	0
2003	0.34	0.27	0.52	0.95	1.66	1.10	0
2004	1.22	1.54	−0.003	0.58	0.04	−0.81	4
2005	0.64	0.79	1.37	1.29	0.94	0.60	4
2006	1.68	2.11	2.91	3.28	3.28	1.15	5
2007^b^	0.48	0.81	0.76	0.92	0.56	0.15	0
2008	1.42	1.58	0.72	0.85	0.06	−0.22	1
2009	1.94	1.52	1.68	2.11	2.34	1.19	0
2010	−0.02	−0.58	0.42	1.57	2.32	1.28	1
2011	0.71	1.16	0.27	0.56	0.13	0.86	1
2012	1.49	1.39	1.36	1.28	0.63	0.79	7
2013	0.61	0.96	0.55	0.76	0.79	1.46	1
2014	1.17	0.61	−0.22	−0.46	−0.22	−0.87	0
2015^b^	−0.30	0.02	−0.25	0.35	−0.09	−0.18	0
2016^b^	−0.18	−0.92	−1.51	−1.44	−0.62	0.00	0
2017^b^	0.25	−0.13	0.30	0.94	0.30	0.07	0
2018	0.86	0.42	0.50	0.15	0.08	1.55	0
2019^c^	2.36	2.30	2.37	2.25	2.54	1.81	12

Abbreviations: EEE, Eastern equine encephalitis; SPI, Standard Precipitation Index.

^a^ The SPI is calculated for a given location based on the long-term precipitation record for the same time period. A positive SPI indicates greater than the median precipitation and a negative SPI, less than the median precipitation.

^b^ For all 6 regions, the SPI was lower than 1.0.

^c^ Data collection ended December 1, 2019.

## Discussion

This cross-sectional study found that a 12-month SPI below 1.0 across Massachusetts was associated with lower incidence of human EEE compared with a 12-month SPI higher than 1.0. A high 12-month SPI measured in July may suggest an increased risk of human EEE cases. Despite these data, estimating the risk of EEE infection is complex and not easily explained simply by precipitation data, and large-scale epidemiologic investigations of EEE are limited by the low number of cases diagnosed each year.^5,6^ Temperature also plays a role in risk of EEE cases, as exemplified by the 2012 data that included 7 human EEE cases. Notably, 2012 was the warmest year in Massachusetts from 1895 through 2012.^7^

This study was limited by the absence of monthly SPI data and monthly data regarding human EEE cases. It is unclear if these results would be found in other states where EEE infections occur, as this study only involved data in Massachusetts.

## Conclusions

The level of precipitation during the life cycle of the enzootic mosquito vector of EEE is an important variable determining the risk of human infection. In the 21st century, as in millennia before, human health cannot be separated from conditions in our environment.
